# Association between lack of dental service utilisation and caregiver‐reported caries in Australian Indigenous children: A national survey

**DOI:** 10.1111/jpc.16192

**Published:** 2022-09-09

**Authors:** Jia R Toh, Nadine Wooi, Si N Tan, Kingsley Wong, Claudia Lopez‐Silva, Sobia Zafar

**Affiliations:** ^1^ School of Dentistry, UQ Oral Health Centre The University of Queensland Brisbane Queensland Australia; ^2^ Child Disability, Telethon Kids Institute University of Western Australia Perth Western Australia Australia

**Keywords:** dental attendance, dental caries, Indigenous children

## Abstract

**Aim:**

To investigate the association between the lack of dental service utilisation and dental caries in Australian Indigenous children.

**Methods:**

Data from the Longitudinal Study of Indigenous Children, which is a longitudinal population‐based cross‐sectional study in Australia were analysed. A total of 1258 children were included, consisting of the baby cohort and the child cohort at Wave 7. Logistic regression analysis was conducted to examine the association between caregiver‐reported child dental caries and dental service utilisation. Multiple imputation using the fully conditional specifications approach was used to account for missing data.

**Results:**

Around one tenth (12.3%) of Indigenous children did not see a dentist when required. Lack of dental service utilisation was associated with an increased likelihood of caregiver‐reported dental caries (odds ratio (OR) 2.4; 95% confidence interval (CI) 1.5–3.8) and teeth removed due to dental caries (OR 2.3; 95% CI 1.1–4.7). These associations remained after adjusting for confounders (caregiver‐reported dental caries OR 2.3; 95% CI 1.3–3.8; teeth removed due to dental caries OR 2.1; 95% CI 1.0–4.4). The reasons reported for not utilising dental services when required were the lack of an available dentist (31.4%), difficulties with physical access (19.8%), long waiting times (13.9%), financial issues with cost (5.8%) and feeling that ‘they could cope’ (4.6%).

**Conclusions:**

Lack of dental service utilisation was associated with dental caries and extraction due to caries in Australian Indigenous children.

## What is already known on this topic

1. Reduced dental service utilisation is broadly associated with poorer oral health and untreated dental caries can cause discomfort, pain, poor sleep, irritability and hospitalisation.
2. Australian Indigenous children experience higher risks of developing dental caries, have greater unmet oral health; and face substantial barriers to accessing dental services compared to non‐Indigenous people.
3. Few studies have investigated the association between dental service utilisation and dental caries, or understood the reasons for reduced dental service utilisation in Australian Indigenous children.

## What this paper adds

1. The lack of dental service utilisation was associated with an increased likelihood of caregiver‐reported dental caries and teeth removed due to dental caries.
2. The shortage of dental treatment providers and geographical remoteness posed as the main barriers to accessing dental services amongst Australian Indigenous children.
3. These findings suitably represent the Australian Indigenous children as a whole population, as the Longitudinal Study of Indigenous Children (LSIC) is the largest national study of Australian Indigenous children to date.

The notion that oral health is integral to one's general health is now becoming more widely recognised.^1^ As such, efforts have been put to advance the oral health of Australians, with strategies such as water fluoridation and improvements to preventative oral health services and oral hygiene education programmes which resulted in a significant reduction in dental caries experience amongst children since the 1970s.^2^

Despite these advances as a nation, oral health disparities have persisted for the Indigenous people. The cumulative effects of colonisation, misguided policies, lack of access to water fluoridation, introduction of the Western diet, and ostracism from education and health care have led to the deterioration of their overall general well‐being and a decline in oral health.^3^ This deterioration has been substantiated by the increased prevalence of preventable, chronic and complex health problems that start early in life.^4^ Such observations are not limited to the Australian Indigenous people, but have also been witnessed in other developed nations, including Canada, New Zealand and America.^5^

This discrepancy has been acknowledged and efforts have been made to close the oral health gap between Indigenous and non‐Indigenous people. The Aboriginal and Torres Strait Islander people are currently recognised as one of four priority populations who experience poorer oral health and are faced with greater barriers to accessing dental services.^1^ Over the past decade, the Australian Government has introduced programmes such as Closing the Gap, Child Dental Benefit Scheme (CDBS), Voluntary Dental Graduate Year Program (VDGYP) as well as the Dental Relocation and Infrastructure support scheme (DRISS), to improve the accessibility of dental services in rural and regional areas.^6^ Despite these developments, Aboriginal and Torres Strait Islander people still face a higher risk of developing dental caries and greater unmet oral health needs compared to non‐Indigenous people.^7^

Poor oral health from untreated dental caries and associated infections can cause discomfort, pain, poor sleep, irritability and in severe acute cases, hospitalisation.^8^ These significantly impact self‐esteem and dietary intake, and more specifically in children, result in interferences with learning and growth.^8^, ^9^, ^10^ As the burden of oral diseases could potentially last a lifetime – with the need for restorative treatment and ongoing maintenance due to restorative failure afterwards, it is imperative to explore and address prospective interventions for a preventative dental approach.^11^

Reduced dental service utilisation has been broadly associated with poorer oral health.^12^, ^13^ Several reasons have been proposed for reduced dental service use including reduced availability of dentists (geographic remoteness), long waiting public dental waiting lists, poor perception of dental service (poorer dental service provided in public), poor oral health literacy (problem‐driven dental appointments) and financial restrictions (posocio‐economic status and lack of dental insurance).^8^, ^12^, ^13^, ^14^, ^15^, ^16^

Although reasons for reduced dental service utilisation in the Australian and paediatric populations have been well‐documented, limited studies have investigated the reasons for reduced dental service utilisation in Australian Indigenous children.^12^, ^13^, ^14^, ^15^ As Australian Indigenous children face a longstanding burden of oral diseases and susceptibility to poor oral health, it is crucial that potential barriers to dental service usage are identified to assist in planning for future oral health interventions. The aim of this study was to investigate the association between dental service utilisation and carer‐reported dental caries in Australian Indigenous children, as well as the reasons for this reduced dental service utilisation in this population group.

## Methods

### Ethical considerations

The LSIC received ethical approval from the Australian Government Department of Health and Ageing Departmental Ethics Committee for the study. This study obtained permission to use the LSIC data set from the National Centre for Longitudinal Data (NCLD) Access for analysis under The University of Queensland's organisational licence.

#### Study population and sampling

This study is a secondary analysis of data from the Longitudinal Study of Indigenous Children (LSIC), which was established in 2008 through a partnership between the Department of Social Services (DSS), the Footprints in Time Steering Committee, and the Australian Institute of Aboriginal and Torres Strait Islander Studies (AIATSIS). LSIC is a nationally representative longitudinal study that collects information about the environment in which Australian Indigenous children grow up in, and the social, economic, educational and family issues that impact their development and well‐being.^17^

The first data set (Wave 1) comprised of a total of 1687 children and was collected in 2008. Follow‐up data were collected every year thereafter, and new participants were added only in Wave 2. A cluster sampling technique was used to select geographic sites. Data were collected from primary caregivers (Parent 1/P1), P1's partner or father (Parent 2/P2) and educators via self‐reported questionnaires. The LSIC study sample identified geographical sites based on Aboriginal and Torres Strait Islander people concentrations and aimed to obtain 150 children from each site. Distribution of participants varied across sites and waves, due to limited population size, participant relocation and withdrawal at different stages of the study. A detailed description of the methodology has been published in the Department of Social Services LSIC data user guide.^17^

#### Data collection

The variable of interest; self‐reported access to dental services, was only reported in Waves 2, 4 and 7. Data from Wave 7 was selected for the cross‐sectional analysis on the basis of recency of information, and the greatest proportion of positive responses. Only information from the primary caregiver (P1) was considered. Participants were stratified into two cohorts: the Baby (B) cohort aged 6.5–8 years old; and the Child (K) Cohort aged 9.5–11 years old at point of data collection in Wave 7.

#### Variables

To investigate the associations between reduced dental service utilisation and self‐reported caries in Australian Indigenous children, the outcome variable was self‐reported dental caries, with the main independent explanatory variable being self‐reported access to dental services reported by P1. Other independent variables were cohort, gender, Indigenous status, oral hygiene habits, daily frequency of sweet food/beverage intake, family construct, socio‐economic status and geographical remoteness.

The outcome variable (carer‐reported caries) was assessed by the question *‘Has study child (SC) ever had any of the following problems with (his/her) teeth or gums – Any cavities, holes or tooth decay?’* and *‘Has SC ever had any of the following problems with (his/her) teeth or gums – tooth pulled out because of decay?’*. Responses to each question were dichotomised into two groups (yes/no). The exposure variable (reduced dental service utilisation) was assessed by the question *‘Has SC ever needed a dentist but didn't see one’* and was dichotomised into two categories (yes/no).

Gender was categorised into male and female. Indigenous status of study child was grouped into three categories: Aboriginal, Torres Strait Islander, and Aboriginal and Torres Strait Islander. To assess oral hygiene habits, the question *‘SC brushes.’* was classified into frequent (twice or more daily) and infrequent (less than twice daily) toothbrushing.^18^, ^19^ As the actual value of sugar consumption could not be accurately measured directly, recommended daily intakes from the current Australian Dietary Guidelines for children were referenced, and the frequency of ‘added sugar’ intake was categorised into ‘Low’ (B: 0, K: 0–2) or ‘High’ (B: 1 or more; K: 3 or more).^20^ As naturally present sugars do not make important contribution to the development of dental caries, only food and beverages with added sugars were included as part of ‘sugar intake’.^21^

For socio‐economic status, decile of the 2006 Index of Relative Indigenous Socioeconomic Outcomes (IRISEO) scores were used and coded into three categories^22^ most disadvantaged (1st–4th deciles), moderately disadvantaged (5th–7th deciles) and least disadvantaged (8th–10th deciles). Caregiver education was assessed by the question ‘*P1 Highest completed qualification*’ and was categorised into four categories based on highest education qualification: (i) Bachelor's degree and higher, (ii) Diploma/TAFE/ Certificate, (iii) Year 12 and (iv) Year 11 or below. The study child's family construct was assessed by the question *‘Study child lives with…’* and was categorised into four categories, (i) parent and partner, (ii) lone parent, (iii) carer and partner and (iv) one carer.

#### Data analysis

The data corresponding to the dependent, independent and confounding variables was identified and downloaded from the LSIC database using IBM SPSS Statistics for Windows v26 (IBM, Armonk, NY, USA). Descriptive statistics, expressed as count (percentage), were used to summarise the characteristics of the study population. Frequency distribution of the variables of interest was reported by caregiver‐reported dental caries and caregiver‐reported reduced dental service utilisation. Logistic regression analysis was conducted to estimate the association of outcome variable (self‐reported dental caries) with the variable of interest (reduced dental service utilisation). An unadjusted analysis was first performed, followed by a multivariable analysis (using the complete case approach) which accounted for the effects of the other independent variables. Finally, a regression analysis with multiple imputation was performed using the fully conditional specifications (FCS) approach. Missing values of diet (frequency of sweet food/beverage intake) and caregiver education were imputed as each had more than 5% missing. Number of imputations was based on the percentage of missing data of both variables (14 imputations, 13.7% missing). All study variables (dental caries, teeth removal, dental service utilisation, cohort, gender, Indigenous status, oral hygiene, family construct and socio‐economic status) were employed to impute the missing values and both logistic (for diet) and ordinal logistic (for education) regressions were used for imputation modelling. To test the departure from missing at random (MAR) assumption, a weighted sensitivity analysis using the selection model approach for the outcome was used. The findings of such analysis confirmed the assumption. Regression estimates were reported as odds ratios (ORs) with 95% confidence intervals (CIs). All statistical analyses were carried out in Stata version 17.0 (StataCorp. 2021. Stata Statistical Software: Release 17. College Station, TX: StataCorp LLC.).

## Results

Socio‐demographic characteristics of the study population are presented in Table 1. The study included 1258 children, of which 734 (58.3%) and 524 (41.7%) were from the baby and child cohort, respectively. Roughly equal proportions of male (49.1%) and female participants (50.9%) were represented in the study sample. The majority of the children were identified as Aboriginal (87.1%), 6.8% as Torres Strait Islander and 6.1% as Aboriginal and Torres Strait Islander. Regarding socio‐economic status, approximately three quarters of the participants fell into moderate (46.6%) and most disadvantaged (30.8%) categories on the IRISEO scale. Approximately one third (36.8%) of mothers had completed a higher education course while the remaining 57.1% had completed high school or less. Nearly half (46.1%) of the children were taken care by either lone parent (40.1%) or lone carer (6.1%), while the remaining children had family constructs including a parent and a partner (50.2%) or carer and their partner (3.2%).

**Table 1:** Distribution of participants' characteristics (*n* = 1258)

Participant characteristics	*n* (%)	Lack of dental service utilisation when needed, *n* (%)
Cohort		
B cohort	734 (58.3)	52 (7.1)
K cohort	524 (41.7)	27 (5.2)
Gender		
Male	618 (49.1)	40 (6.5)
Female	640 (50.9)	39 (6.1)
Indigenous status		
Aboriginal	1096 (87.1)	64 (5.8)
Torres Strait Islander	85 (6.8)	9 (10.6)
Aboriginal and Torres Strait Islander	77 (6.1)	6 (7.8)
Caregiver‐reported decay		
Yes	377 (30.0)	39 (10.3)
No	869 (69.1)	40 (4.6)
Invalid response	12 (1.0)	0 (0)
Ever had teeth removed because of decay		
Yes	79 (6.3)	10 (12.7)
No	1167 (92.8)	69 (5.9)
Invalid response	12 (1.0)	0 (0)
Oral hygiene		
Infrequent, <2× daily	815 (64.8)	54 (6.6)
Frequent, ≥2× daily	435 (34.6)	25 (5.7)
Invalid response	8 (0.6)	0 (0)
Frequency of sweet food/beverage intake (per day)		
Low (B: 0; K 0–2)	650 (51.7)	30 (4.6)
High (B: 1 or more; K: 3 or more)	501 (39.8)	37 (7.4)
Invalid response	107 (8.5)	12 (11.2)
Socio‐economic status 2006 Decile of Relative Indigenous Socioeconomic Outcomes (IRISEO)		
Most disadvantaged (1–4)	388 (30.8)	35 (9.0)
Moderate (5–7)	586 (46.6)	31 (5.3)
Least disadvantaged (8–10)	279 (22.2)	13 (4.7)
Invalid response	5 (0.4)	0 (0)
Parental education		
Bachelor's degree or higher	107 (8.5)	5 (4.7)
Diploma/TAFE/Certificate	356 (28.3)	13 (3.6)
High School (Year 12)	178 (14.1)	10 (5.6)
Year 11 or below	541 (43.0)	42 (7.7)
Invalid response	76 (6.0)	3 (3.9)
Family construct		
Parent and partner	631 (50.2)	40 (6.3)
Lone parent	505 (40.1)	30 (5.9)
Carer and partner	40 (3.2)	1 (2.5)
Lone carer	77 (6.1)	8 (10.4)
Invalid response	5 (0.4)	0 (0)

In terms of oral hygiene, 64.8% of children had performed oral hygiene less than twice daily while 34.6% performed oral hygiene twice or more daily. As for sugar consumption, the highest percentage of children (51.7%) was evident in the group with low frequency of sweet food or beverage intake in a given day. Furthermore, on dental service utilisation, slightly more than one tenth (12.3%) of parents reported their children not utilising dental services when needed. Multiple reasons for this were selected by their caregivers. In descending order, 31.4% reported having the lack of an available dentist as a barrier, 19.8% reported having transportation or distance barriers, 13.9% reported long waiting times affecting access to dental services, 5.8% reported cost as a barrier, 4.6% reported not utilising dental services as they ‘felt they could cope’. The option ‘others’ was also explored, with 24.4% of participants selecting this option. No participants responded to ‘another carer didn't take the child’, ‘dislikes the service or staff’, ‘discrimination’, ‘language problems’ or ‘someone else dealt with the problem’ as a reason for not utilising dental services when needing one (Table 1).

Indigenous children who did not utilise dental services when required showed higher prevalence of caregiver‐reported caries compared to those who utilised dental services when needed (49.4% and 28.9%, respectively). Similarly, Indigenous children who did not utilise dental services when needed also showed higher prevalence of having teeth extracted due to caries compared to those who utilised dental services when needed (12.7% and 5.9%, respectively). Higher prevalence of caregiver reported caries and teeth removed due to dental caries were also observed in four groups: (i) had poor oral hygiene, (ii) high sugar consumption frequency, (iii) low‐income backgrounds and (iv) lone carer family constructs (Table 2).

**Table 2:** Proportion of caregiver reported dental caries and teeth removed due to caries in Australian Indigenous children

	Caregiver‐reported dental caries		Teeth removed due to dental caries	
	(*n* = 1246)		(*n* = 1246)	
	Yes (*n* = 377)	No (*n* = 869)	Yes (*n* = 79)	No (*n* = 1167)
	*n* (%)	*n* (%)	*n* (%)	*n* (%)
Cohort				
B cohort	228 (31.2)	503 (68.8)	54 (7.4)	677 (92.6)
K cohort	149 (28.9)	336 (71.1)	25 (4.8)	490 (95.2)
Gender				
Male	179 (29.3)	432 (70.7)	41 (6.7)	570 (93.3)
Female	198 (31.2)	437 (68.8)	38 (6.0)	597 (94.0)
Indigenous status				
Aboriginal	347 (32.0)	738 (68.0)	73 (6.7)	1012 (93.3)
Torres Strait Islander	13 (15.5)	71 (84.5)	3 (3.6)	81 (96.4)
Aboriginal and Torres Strait Islander	17 (22.1)	60 (77.9)	3 (3.9)	74 (96.1)
Socio‐economic status				
Education level	23 (21.5)	84 (78.5)	4 (3.7)	103 (96.3)
Bachelor's degree or higher	107 (30.1)	248 (69.9)	22 (6.2)	333 (93.8)
Diploma/TAFE/Certificate	42 (23.7)	135 (76.3)	10 (5.7)	167 (94.3)
High School (Year 12) Year 11 or below	183 (34.0)	355 (66.0)	38 (7.1)	500 (92.9)
Income				
Most disadvantaged (1–4)	135 (35.1)	250 (64.9)	35 (9.1)	350 (90.9)
Moderate (5–7)	179 (30.8)	403 (69.2)	30 (5.2)	552 (94.8)
Least disadvantaged (8–10)	63 (22.6)	216 (77.4)	14 (5.0)	265 (95.0)
Family construct				
Parent and partner	182 (28.9)	447 (71.1)	41 (6.5)	588 (93.5)
Lone parent	165 (32.8)	338 (67.2)	31 (6.2)	472 (93.8)
Carer and partner	7 (17.5)	33 (82.5)	2 (5.0)	38 (95.0)
Lone carer	23 (31.1)	51 (68.9)	5 (6.8)	69 (93.2)
Oral hygiene				
Frequent brushing	116 (26.7)	318 (73.3)	21 (4.8)	413 (95.2)
Infrequent brushing	260 (32.1)	550 (67.9)	58 (7.2)	752 (92.8)
Frequency of sweet food/beverage intake (per day)				
Low	179 (27.7)	467 (72.3)	35 (5.4)	611 (94.6)
High	164 (32.8)	336 (67.2)	36 (7.2)	464 (92.8)
Lack of utilisation of dental care when needed				
Yes	39 (49.4)	40 (50.6)	10 (12.7)	69 (87.3)
No	337 (28.9)	827 (71.1)	69 (5.9)	1095 (94.1)

Results from the regression analysis are presented in Table 3. The lack of dental service utilisation when needed was associated with an increased likelihood of caregiver‐reported dental caries (unadjusted OR 2.4, 95% CI 1.5–3.8) and teeth removal due to dental caries (unadjusted OR 2.3, 95% CI 1.1–4.7), and the effects remained after adjusting for confounding factors in the model with multiple imputation (Model 3) (dental caries: adjusted OR 2.4, 95% CI 1.5–3.8; teeth removal: adjusted OR 2.1, 95% CI 1.0–4.3).

**Table 3:** Associations between the lack of dental service utilisation and self‐reported caries/teeth removed due to dental caries in Australian Indigenous children

		Model 1 (*n* = 1243)		Model 2 (*n* = 1087)		Model 3 (*n* = 1241)	
		OR	95% CI	OR	95% CI	OR	95% CI
Caregiver‐reported dental caries							
Yes	39/337†	2.4	1.5–3.8	2.1	1.2–3.7	2.4	1.5–3.8
No	40/827†						
Teeth removed due to dental caries							
Yes	10/69†	2.3	1.1–4.7	1.9	0.8–4.5	2.1	1.0–4.3
No	69/1095†						

† Number of children with reduced dental service utilisation/number of children without reduced dental service utilisation.

CI, confidence interval; OR, odds ratio.

Model 1: Unadjusted.

Model 2: Adjusted for cohort, gender, aboriginal status, diet, oral hygiene, parental education, family construct and socio‐economic status (complete case only).

Model 3: Adjusted for cohort, gender, aboriginal status, diet, oral hygiene, parental education, family construct and socio‐economic status (multiple imputations).

## Discussion

In summary, it was observed an association of the lack of dental service utilisation with both caregiver‐reported dental caries and teeth extracted due to dental caries in Australian Indigenous children. These associations were found to be present after adjusting for confounding factors including cohort, gender, Indigenous status, oral hygiene habits, daily frequency of sweet food/beverage intake, family construct, socio‐economic status and geographical remoteness.

These findings supplement the growing body of evidence on the negative impact of lack of dental service utilisation amongst the Australian Indigenous population. These findings are congruent with other previously completed studies where Indigenous populations were found to have higher DMFT scores.^7^, ^23^ It is attributed to a plethora of reasons such as geographical limitations, lack of available health‐care providers, long waiting times for oral health‐care services, family's financial limitations, family constructs and cultural barriers.^3^, ^23^, ^24^

Further examination of the reasons for the lack of dental service utilisation explained that majority of the respondents within the study responded having the ‘*lack of available dentist’* as the primary barrier in seeking dental services. Despite the Australian Government Department of Health reporting a compound annual growth rate in the dental workforce in 2019, a disproportionate allocation of dental practitioners between metropolitan regions and remote areas was noted, with half as many dental practitioners (full‐time equivalent) distributed in remote and very remote areas in comparison to major cities.^25^ In addition, the geographical remoteness of Indigenous Australians also poses logistical challenges to both the construction and maintenance of required oral health‐care infrastructures.^26^ These logistical constraints, coupled with staffing shortages, geographical distance and/or equipment failure, can limit supply of dental service provision in remote and regional areas, stressing the operating capacities of existing dental service providers and resulting in longer waiting times for treatment.^27^ Financial barriers to accessing dental services were also cited in the LSIC. The socio‐economic disadvantage of the Australian Indigenous population can limit access to dental services, which exacerbates the development and implications of dental caries.^24^ Socio‐economic factors such as caregiver education, household financial hardship, household overcrowding, parents' occupation and employment status have been reported to affect the capacity of Australian Indigenous children to afford private dental services.^28^ The Australian Government has implemented strategies to curb financial barriers such as the Child Dental Benefits Schedule (CDBS) which provides benefits for dental services, and the Dental Relocation and Infrastructure Scheme (DRISS), which provides relocation and infrastructure support grants for dental providers. While these schemes have made private dental services more available, Australian Indigenous children are still face challenges in utilising and accessing these targeted dental initiatives.^29^, ^30^ Factors that are considered to influence this include distance and remoteness,^5^ lack of knowledge of the services available^31^ and limited integration of culturally safe practice.^32^

It is thus important to acknowledge that while there were no participants who responded to the discrimination or language options as barriers, a quarter of respondents indicated there were ‘other’ barriers to access without further elaboration. Several other studies have put forward potential reasons for reduced dental service use amongst Australian Indigenous children, including family and cultural level influences.

Family constructs with single parents or carers, have been identified as a risk factor for dental caries in children due to family stresses arising from parenting responsibilities and reduced personal resources related to separation and family conflict.^33^ Restricted finances and lack of partner support, coupled with the opportunity cost of missing work for their children's dental appointments can subsequently hinder the ability of these children to access dental services.^33^ Cultural misconceptions such as ‘poor oral health being natural’, ‘oral hygiene being non‐essential’, and notions that ‘primary teeth being less important than permanent teeth’ present can also impair motivation to seek for dental services.^34^ In addition, Indigenous Australians also engage in problem‐based dental attendance patterns.^35^ This delayed response to dental problems can subsequently lead to the need for more invasive dental treatment,^36^ compared to their non‐Indigenous counterparts.^35^

One major strength of this study is that LSIC is the largest national study of Australian Indigenous children to date, with study sites selected amongst all of Australia's states and territories, inclusive of urban, regional and remote areas.^37^ Hence, it is a suitable representation of the Australian Indigenous children population. In addition, the LSIC also included participants across a diverse range of life circumstances, location and cultures – which enabled a more precise depiction of the Aboriginal and Torres Strait Islander people's life.^38^ Additionally, results found were adjusted for consistent confounding factors reported within the LSIC dataset including cariogenic diets, oral hygiene, family construct, socio‐economic status and caregiver education.^18^

The biggest limitation of this study is that it is a secondary analysis of the LSIC dataset. As such, the results are bounded by the constructs of the LSIC dataset, and the authors were unable to control for these limitations. One other limitation is that the study outcomes (dental caries and teeth extracted) were reported by caregivers and not evaluated clinically possibly leading to potential under‐reporting and underestimation of dental caries.^39^ However, it has been demonstrated that caregiver‐reporting proves to be a reliable method of data collection when collection of clinical data is restricted due to cost or logistics.^40^ Another limitation is that the LSIC adopted a non‐probability sampling method during the initial recruitment process. Nevertheless, it is still important to acknowledge that the Aboriginal and Torres Strait Islander peoples share similar general characteristics, and that the LSIC study is not intended to be a representative of all Aboriginal and Torres Strait Islander families but rather, to provide a snapshot of life in a diverse range of environments.^37^, ^41^

Within the constructs of this study, the shortage of dental treatment providers and geographical remoteness posed as the main barriers to accessing dental services amongst Australian Indigenous children. However, due to the close‐ended design of the LSIC study, not all reasons for reduced dental service utilisation could be accurately evaluated. More studies involving clinically measured outcomes for an objective assessment of oral health conditions including caries, and open‐ended questionnaires to facilitate a qualitative analysis are required to better understand the relationship between the lack of dental service utilisation and oral health outcomes in Australian Indigenous children.

## Conclusions

This study confirms that the lack of dental service utilisation when required is associated with caregiver‐reported dental caries and teeth removed due to caries in Australian Indigenous children. Findings from this study could potentially contribute to future policy planning, in terms of creating effective oral health promotion and dental service utilisation programmes with emphasis primary prevention of caries and its adverse outcomes among Australian Indigenous children.
